# Multifocal demyelinating motor neuropathy and hamartoma syndrome associated with a de novo *PTEN* mutation

**DOI:** 10.1212/WNL.0000000000005566

**Published:** 2018-05-22

**Authors:** Boglarka Bansagi, Vietxuan Phan, Mark R. Baker, Julia O'Sullivan, Matthew J. Jennings, Roger G. Whittaker, Juliane S. Müller, Jennifer Duff, Helen Griffin, James A.L. Miller, Grainne S. Gorman, Hanns Lochmüller, Patrick F. Chinnery, Andreas Roos, Laura E. Swan, Rita Horvath

**Affiliations:** From the Wellcome Centre for Mitochondrial Research (G.S.G.), Institute of Genetic Medicine (B.B., M.J., J.S.M., J.D., H.G., H.L., P.F.C., A.R., R.H.), and Institute of Neuroscience (M.R.B., R.G.W., G.S.G.), Newcastle University, Newcastle upon Tyne, UK; Leibniz-Institute für Analytische Wissenschaften-ISAS-e.V. (V.P., A.R.), Dortmund, Germany; Departments of Neurology (M.R.B., J.A.L.M., G.S.G.) and Clinical Neurophysiology (M.R.B., R.G.W., R.H.), Royal Victoria Infirmary, Newcastle upon Tyne; Department of Cellular and Molecular Physiology (J.O., L.E.S.), Institute of Translational Medicine, University of Liverpool; Department of Clinical Neurosciences (P.F.C.), University of Cambridge, Cambridge Biomedical Campus, UK; Department of Neuropediatrics and Muscle Disorders (H.L.), Medical Center – University of Freiburg, Faculty of Medicine, Freiburg, Germany; and Centro Nacional de Análisis Genómico (CNAG-CRG) (H.L.), Center for Genomic Regulation, Barcelona Institute of Science and Technology (BIST), Barcelona, Spain.

## Abstract

**Objective:**

To describe a patient with a multifocal demyelinating motor neuropathy with onset in childhood and a mutation in phosphatase and tensin homolog (*PTEN*), a tumor suppressor gene associated with inherited tumor susceptibility conditions, macrocephaly, autism, ataxia, tremor, and epilepsy. Functional implications of this protein have been investigated in Parkinson and Alzheimer diseases.

**Methods:**

We performed whole-exome sequencing in the patient's genomic DNA validated by Sanger sequencing. Immunoblotting, in vitro enzymatic assay, and label-free shotgun proteomic profiling were performed in the patient's fibroblasts.

**Results:**

The predominant clinical presentation of the patient was a childhood onset, asymmetric progressive multifocal motor neuropathy. In addition, he presented with macrocephaly, autism spectrum disorder, and skin hamartomas, considered as clinical criteria for PTEN-related hamartoma tumor syndrome. Extensive tumor screening did not detect any malignancies. We detected a novel de novo heterozygous c.269T>C, p.(Phe90Ser) *PTEN* variant, which was absent in both parents. The pathogenicity of the variant is supported by altered expression of several PTEN-associated proteins involved in tumorigenesis. Moreover, fibroblasts showed a defect in catalytic activity of PTEN against the secondary substrate, phosphatidylinositol 3,4-trisphosphate. In support of our findings, focal hypermyelination leading to peripheral neuropathy has been reported in PTEN-deficient mice.

**Conclusion:**

We describe a novel phenotype, PTEN-associated multifocal demyelinating motor neuropathy with a skin hamartoma syndrome. A similar mechanism may potentially underlie other forms of Charcot-Marie-Tooth disease with involvement of the phosphatidylinositol pathway.

Inherited peripheral neuropathies (Charcot-Marie-Tooth disease [CMT]) encompass genetically heterogeneous sensory or motor nerve conditions.^1^ Next generation sequencing^2^ and proteomics^3^ are powerful and robust methodologic approaches to elucidate the underlying molecular cause and pathologic mechanisms.

Phosphatase and tensin homolog located on chromosome 10 (*PTEN*) is a tumor suppressor gene in which mutations are typically found in sporadic cancers and inherited tumor susceptibility conditions, such as the PTEN hamartoma tumor syndrome (PHTS). Macrocephaly, autism spectrum disorder (ASD), ataxia, tremor, and epilepsy have been reported in patients with *PTEN* mutations, while functional implications of this protein have been investigated in Parkinson and Alzheimer diseases.^4,5^ Various PTEN functions in neurons have been linked to its dynamic spatiotemporal distribution. Regulation of neuronal cell size, axonal outgrowth, synaptogenesis, and neuromuscular assembly are PTEN-controlled processes during neuronal development and regeneration after injury. Furthermore, in PTEN-deficient mice abnormal regulation of myelination has been observed in peripheral nerves.^6,7^

Here we describe a patient manifesting with multifocal demyelinating motor neuropathy and hamartoma syndrome in association with a de novo heterozygous *PTEN* mutation.

## Methods

A 29-year-old patient had been born to nonconsanguineous Caucasian parents. Delayed motor development and speech were reported with facial asymmetry at 7 years of age. He developed focal weakness involving his right eye movements, his face, and contralateral extremities in the following years. By age 20, multiple livid papules and keloid scars appeared on his chest and he had bowel problems and learning difficulties.

Examination at age 29 revealed a large scaphocephalic head (macrocephaly), asymmetric face, and crowded teeth. The patient had upper thoracic scoliosis. Right oculomotor palsy, complete right-sided and partial left-sided facial palsy, and left-sided tongue atrophy were noted. He had nasal speech. There was both proximal and distal flaccid paresis in the left upper limb, with atrophy and areflexia. In the right arm, he had proximal flexor weakness but preserved distal grip strength. The right leg was short with pes cavus and hammertoes along with distal motor weakness and atrophy. The left leg showed no wasting and only mildly decreased strength in foot extensors. Knee jerk reflexes were brisk bilaterally, but ankle jerk reflexes were absent. Sensory examination was normal. There was no cerebellar ataxia, but he had learning difficulties.

**Figure 1:**
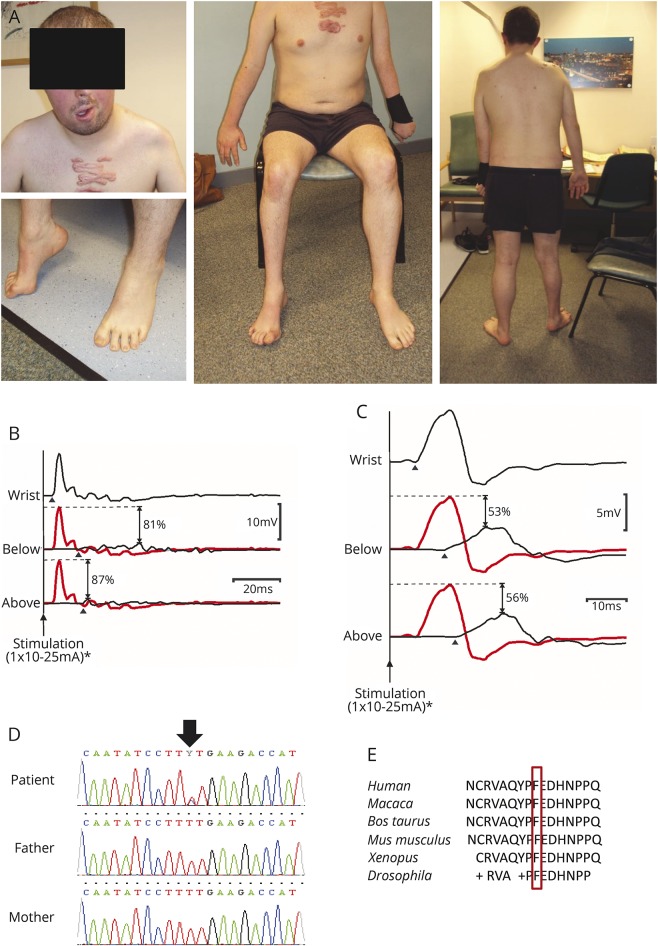
Clinical and electrophysiologic features (A) Photographs of the patient show the asymmetric focal neuropathy. (B) Right abductor pollicis brevis (APB) compound muscle action potentials (CMAPs) obtained by stimulating the median nerve at the wrist (top), the antecubital fossa (middle), and above the elbow (bottom) demonstrate significant conduction block (with desynchronization) affecting the right median nerve within the forearm. In the lower traces, proximal responses (black lines) are superimposed on the distal CMAP (plotted in gray), thus facilitating visualization of the conduction block at each location (the percentage drop in CMAP amplitude at each site is also indicated). (C) Right abductor digiti minimi (ADM) CMAPs evoked by stimulating the ulnar nerve at the wrist (top) and below (middle) and above (bottom) the medial epicondyle demonstrate significant conduction block (with dispersion) affecting the right ulnar nerve within the forearm. Maximal distal CMAPs were obtained using stimulus currents of ∼10 mA and to confirm conduction block currents of ∼25 mA were used at proximal locations (pulse duration 0.5 ms). Stimulus artefacts have been removed for clarity. Arrowheads mark CMAP onset. *Significantly higher stimulus currents (up to 100 mA) were used to confirm conduction block during clinical testing. (D) Electropherogram shows the de novo heterozygous c.269T>C, p.(Phe90Ser) *PTEN* mutation. (E) Evolutionary conservation of the mutation.

Nerve conduction studies showed markedly reduced asymmetric compound muscle action potential responses with dispersion and conduction slowing and significant conduction block at multiple sites fulfilling the AAEM 2003 criteria for definite multifocal motor neuropathy (figure 1, B and C). Sensory responses were within acceptable limits throughout. On EMG, neurogenic changes were observed in the wasted limbs but no evidence of recent denervation was found except in right deltoid on the most recent EMG requested after the patient complained of new onset shoulder weakness (tables e-1–e-4, links.lww.com/WNL/A481).

Imaging and laboratory studies including CSF were repeatedly unremarkable. Routine genetic diagnostic testing revealed normal CGH array and no pathogenic variants were detected on the next-generation panel for 56 genes associated with CMT.^2^ Immunosuppressive therapy was tried without benefit.

We performed whole exome sequencing in the patient's genomic DNA.^2^ Variants were annotated and filtered against protein prediction, evolutionary conservation, and frequency in several databases and were validated by Sanger sequencing and segregation analysis in the patient and in his parents with special attention on variants in all known CMT-related genes. Immunoblotting was performed in fibroblasts by standard techniques. Enzyme function test was carried out using in vitro malachite green phosphatase assay as described.^7^ Label-free shotgun proteomic profiling utilizing patient-derived (4 biological replicates) and control fibroblasts (6 biological replicates) was performed (e-Methods, links.lww.com/WNL/A482).

### Data availability

Whole exome sequencing data of the patient has been deposited in RD-CONNECT (rd-connect.eu/) and in the European Genome Phenome Archive (ega-archive.org/). The mass spectrometry data has been deposited to the ProteomeXchange Consortium31 via the PRIDE partner repository with data identifier PXD008014 (proteomecentral.proteomexchange.org/cgi/GetDataset).

## Results

The patient carried a novel de novo heterozygous c.269T>C, p.(Phe90Ser) *PTEN* variant, which was absent in both parents (figure 1D). The mutation has not been reported in the ExAc database and it affects a highly conserved amino acid residue within the catalytic site of PTEN phosphatase domain and was predicted to be deleterious.

**Figure 2:**
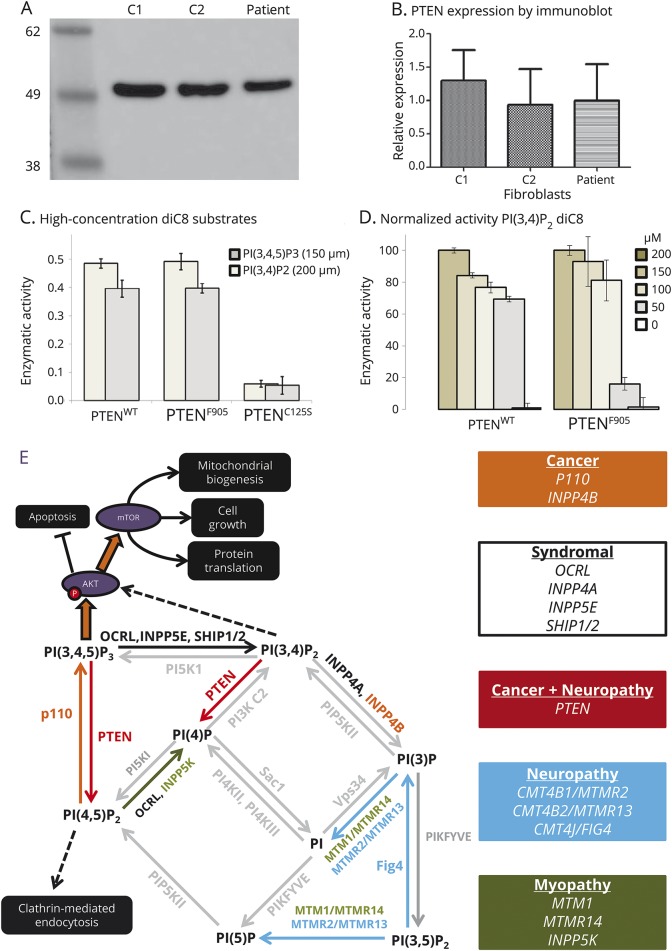
Functional effects of abnormal phosphatase and tensin homolog located on chromosome 10 (PTEN) (A) Immunoblot (with marker size in kDa) shows expression of PTEN in fibroblasts from 2 controls (C1, C2) and the patient. (B) Quantification of mean relative PTEN expression between controls (C1, C2) and patient fibroblasts (3 replicates). (C) PTEN^C124S^ but not PTEN^F90S^ shows reduced enzymatic activity against 150 µM PI(2,4,5)P_3_ and 200 µM PI(3,4)P_2_ compared to PTEN^WT^. (D) PTEN^F90S^ shows reduced activity against <100 µM PI(3,4)P_2_ compared to PTEN^WT^. (E) The roles of PTEN within the phosphatidylinositol system, with human mutations causing neuropathy and cancer highlighted according to the key.

Immunoblotting revealed normal levels of PTEN in the patient's fibroblasts (figure 2, A and B). Enzyme function assay revealed no impaired p.(Phe90Ser) PTEN phosphatase activity on phosphatidylinositol 3,4,5-trisphosphate (PIP_3_) as a substrate when compared the wild-type protein. However, there was a defect in catalytic activity against the secondary substrate of PTEN, phosphatidylinositol 3,4-trisphosphate (PI[3,4]P_2_), which was no longer apparent at high levels of PI(3,4)P_2_ (figure 2, C and D).

**Figure 3:**
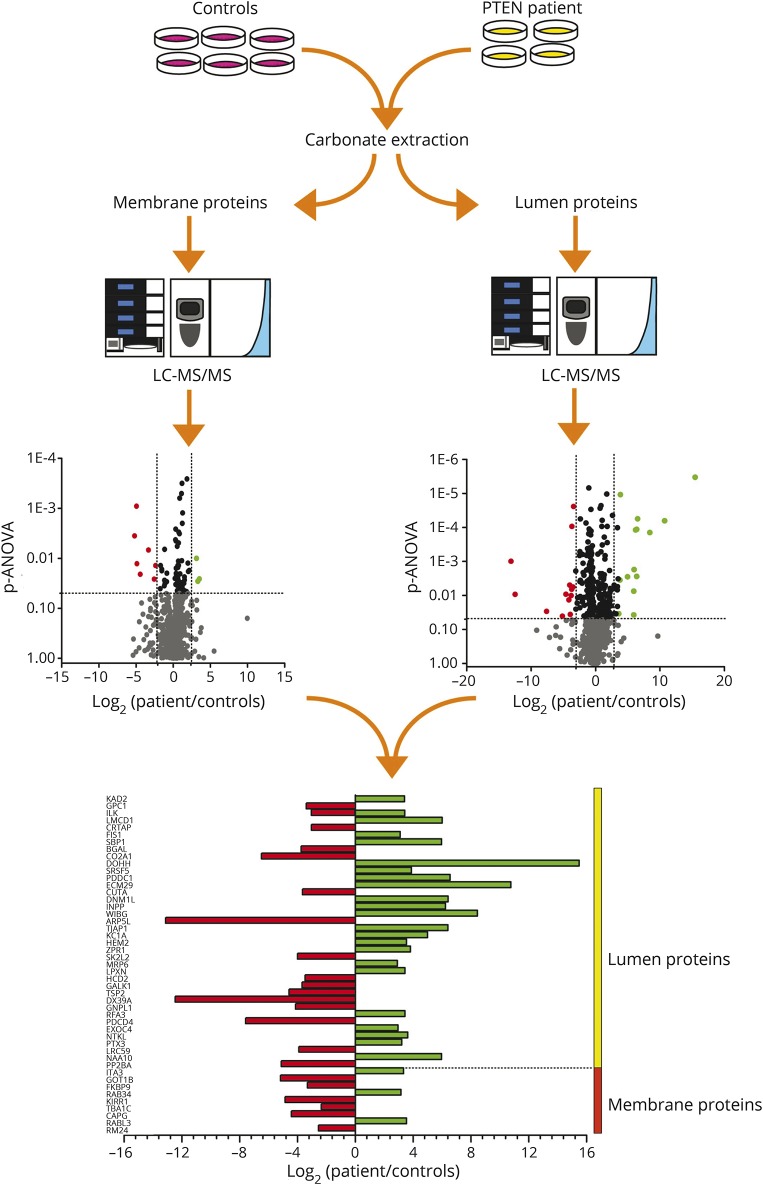
Proteomic profiling Proteomic profiling in patient-derived fibroblasts revealed a total of 1,944 distinct proteins with altered abundance of 47 proteins.

Proteomic profiling in patient-derived fibroblasts revealed a total of 1,944 distinct proteins with altered abundance of 47 (9 proteins based on 2 or more peptides, 38 proteins on one unique peptide) with different subcellular localizations such as cytosol and cell membrane (figure 3 and figure e-1, links.lww.com/WNL/A480; e-Methods, links.lww.com/WNL/A482). LMCD1 and ITA3 are associated with cell migration and metastasis formation,^8^ while AK2, ILK, and SBP1 are involved in cellular antitumor response.^9,10^

## Discussion

Germline *PTEN* mutations cause various human conditions with poorly understood genotype–phenotype correlations.^4,11^ *PTEN* shows limited genetic variation in the human population (1000 Genome Project found 54 single nucleotide variants with minor allele frequency <0.001) and the protein is highly conserved between species. Cytosolic PTEN consists of 403 amino acids divided into an N-terminal phosphatase domain (7–185), a lipid-binding C2 domain (186–351), and a C-terminal tail (352–403), containing phosphorylation sites that regulate protein activity and stability.^11^ PTEN directly antagonizes Class I phosphoinositide 3-kinases (PI[3]Ks) by converting PIP_3_ into phosphatidylinositol 4,5-bisphosphate (PI[4,5]P_2_). Diminished PI(3)K signaling exerts a brake on downstream effector pathways interfering with cellular processes such as cell growth, proliferation, and survival.^5,11^ The alternate PTEN substrate, PI(3,4)P_2_, has many similar signaling functions to PIP_3_ and several tumors are caused by preferential loss of hydrolysis of PI(3,4)P_2_ substrates^12^ and depending on its subcellular location PI(3,4)P_2_ is both a suppressor and enhancer of mTOR signaling.^13^

Loss of function germline mutations in *PTEN* are mainly associated with inherited tumors and severe PHTS, while retained or partially lost PTEN activity has been found in neurodevelopmental disorders.^4^ Besides its dual-specific phosphatase activity, phosphatase-independent mechanisms and protein–protein interactions have also been implicated in *PTEN* mutations.^5^

The p.(Phe90Ser) missense change identified in our patient is located within the WPD loop, which is equally near to the phosphatase active pocket and to the membrane-binding regulatory interface of the PTEN C2 domain. Most of the mutated WPD residues have been considered not to influence or to only partially affect PTEN catalytic activity.^14^ The somatic p.(Phe90Ser) *PTEN* mutation has been found in cancer (glioma, endometrium), and had normal phosphatase activity against PIP_3_ but a reduced ability to bind membrane phospholipids and recruit PTEN for PIP_3_ signalling.^7^ The germline c.269T>C, p.(Phe90Ser) *PTEN* mutation in fibroblasts of our patient produced a stable protein with an unexpected deficit in activity against PI(3,4)P_2_. This may be due to a novel allostery in the mutant or a change in the vulnerability of the protein to oxidation, which inhibits PTEN activity in these assays. In agreement with our data, a recent publication confirmed that PTEN is a PI(3,4)P_2_ 3-phosphatase and together with INPP4B regulates PI(3,4)P_2_ accumulation downstream of Class I PI3K, and PI(3,4)P_2_ signaling may play a role in the tumor suppressor function of PTEN.^15^

The phenotype of this patient was unique and different from the neurologic presentations associated with PTEN defects reported previously. Extensive tumor screening did not detect any malignancies, but we considered his hypertrophic keloids as skin hamartomas. While fulfilling some of the clinical criteria of PHTS, including the presence of macrocephaly, skin lesions, and ASD, the predominant feature at presentation was an asymmetric, gradually progressive multifocal demyelinating motor neuropathy, with electrophysiologic evidence of definite partial conduction block fulfilling American Academy of Electrodiagnostic Medicine diagnostic criteria for multifocal motor neuropathy.^16^ A susceptibility to demyelination following mild trauma—similar to hereditary neuropathy with liability to pressure palsies (HNPP)—might explain the multifocal presentation in this disorder. In support of this theory, in a mouse model with targeted mutation of *PTEN* in Schwann cells, progressive peripheral neuropathy with focal hypermyelination, myelin outfoldings, and “tomacula” formation has been reported, showing striking similarities to the pathologic findings in HNPP.^6^ A similar clinical presentation with rapidly progressive asymmetric demyelinating neuropathy with conduction blocks and temporal dispersion affecting one limb, resembling an acquired inflammatory neuropathy, has been described in a patient with a *FIG4* mutation, another rare cause of inherited demyelinating related to abnormal levels of PI 3-phosphates.^17^ Furthermore, transheterozygous PTEN mutant *Drosophila melanogaster* have been found to have progressive motor function loss, with deficits in climbing and flight ability.^18^

Several genetic forms of CMT have been described with abnormal myelin histopathology and tomacula formation (CMT4B, CMT4H, CMT4F, CMT1B, and HNPP).^1^ The causative proteins contain phosphoinositide binding domains in CMT2B/RAB7, CMT4B1/MTMR2, CMT4B2/SBF2, CMT4J/FIG4, and CMT4H/FGD4, suggesting that abnormal PI(3)K signaling is a common final pathway in demyelinating CMT (figure 2E). Changes in phosphoinositide metabolites can have many possible cellular effects interfering with normal myelin maintenance and the enzymes involved or their downstream targets are appealing therapeutic targets. Selective inhibition of PI(3)K is a novel anticancer therapy approach already in clinical trials with favorable toxicity profile.^19^ Metabolomic studies of the same inhibitor show that plasma metabolites can be useful dose-dependent biomarkers of the response to therapy.^20^

Results of our proteomic profiling allowed the identification of several proteins supporting abnormal PTEN function: increased LMCD1 has been linked to tumor metastasis^8^ and ITA3 is involved in promoting cell invasion.^21^ In addition, we detected increased AK2, a negative regulator of tumor cell growth,^9^ indicating a compensatory effect, and may explain why extensive tumor screen did not detect malignant processes in our patient. In the same context, increased SBP1 may act as a tumor suppressor.^10^ Alteration of ITA3 and ILK suggests that integrin stimulation is a downstream effect of PTEN.^22^ We found several proteins, including GPC1, which are known to be regulated by PTEN activity and linked to peripheral myelination and axon guidance.

Our data revealed changes in expression of several proteins involved in tumorigenesis or tumor suppression, supporting a pathogenic role for the *PTEN* mutation, which may have caused the focal demyelinating motor neuropathy in association with a cutaneous hamartoma syndrome in our patient. We detected a concentration-dependent defect in catalytic activity of PTEN against its secondary substrate, PI(3,4)P_2_, which may lead to multifocal demyelinating motor neuropathy in *PTEN* mutations and potentially in other CMTs with similar pathomechanisms.

## Glossary

ASD: autism spectrum disorder
CMT: Charcot-Marie-Tooth disease
HNPP: hereditary neuropathy with liability to pressure palsies
PHTS: PTEN hamartoma tumor syndrome
PI(3)K: phosphoinositide 3-kinase
PI(3,4)P_2_: phosphatidylinositol 3,4,-trisphosphate
PIP_3_: phosphatidylinositol 3,4,5-trisphosphate
PTEN: phosphatase and tensin homolog located on chromosome 10

## References

[1] Rossor AM, Evans MR, Reilly MM. A practical approach to the genetic neuropathies. Pract Neurol 2015;15:187–198. 25898997 10.1136/practneurol-2015-001095

[2] Bansagi B, Griffin H, Whittaker RG, et al. Genetic heterogeneity of motor neuropathies. Neurology 2017;88:1226–1234. 28251916 10.1212/WNL.0000000000003772 PMC5373778

[3] Weis J, Claeys KG, Roos A, et al. Towards a functional pathology of hereditary neuropathies. Acta Neuropathol 2017;133:493–515. 27896434 10.1007/s00401-016-1645-y

[4] Spinelli L, Black FM, Berg JN, Eickholt BJ, Leslie NR. Functionally distinct groups of inherited PTEN mutations in autism and tumour syndromes. J Med Genet 2015;52:128–134. 25527629 10.1136/jmedgenet-2014-102803 PMC4316932

[5] Kreis P, Leondaritis G, Lieberam I, Eickholt BJ. Subcellular targeting and dynamic regulation of PTEN: implications for neuronal cells and neurological disorders. Front Mol Neurosci 2014;7:23. 24744697 10.3389/fnmol.2014.00023 PMC3978343

[6] Goebbels S, Oltrogge JH, Wolfer S, et al. Genetic disruption of Pten in a novel mouse model of tomaculous neuropathy. EMBO Mol Med 2012;4:486–499. 22488882 10.1002/emmm.201200227 PMC3443946

[7] Nguyen HN, Yang JM Jr, Rahdar M, et al. A new class of cancer-associated PTEN mutations defined by membrane translocation defects. Oncogene 2015;34:3737–3743. 25263454 10.1038/onc.2014.293 PMC4377315

[8] Chang CY, Lin SC, Su WH, Ho CM, Jou YS. Somatic LMCD1 mutations promoted cell migration and tumor metastasis in hepatocellular carcinoma. Oncogene 2012;31:2640–2652. 21996735 10.1038/onc.2011.440

[9] Kim H, Lee HJ, Oh Y, et al. The DUSP26 phosphatase activator adenylate kinase 2 regulates FADD phosphorylation and cell growth. Nat Commun 2014;5:3351. 24548998 10.1038/ncomms4351 PMC3948464

[10] Ansong E, Ying Q, Ekoue DN, et al. Evidence that selenium binding protein 1 is a tumor suppressor in prostate cancer. PLoS One 2015;10:e0127295. 25993660 10.1371/journal.pone.0127295 PMC4436248

[11] Leslie NR, Longy M. Inherited PTEN mutations and the prediction of phenotype. Semin Cel Dev Biol 2016;52:30–38. 10.1016/j.semcdb.2016.01.030 26827793

[12] Fedele CG, Ooms LM, Ho M, et al. Inositol polyphosphate 4-phosphatase II regulates PI3K/Akt signaling and is lost in human basal-like breast cancers. Proc Nat Acad Sci USA 2010;107:22231–22236. 21127264 10.1073/pnas.1015245107 PMC3009830

[13] Marat AL, Wallroth A, Lo WT, et al. mTORC1 activity repression by late endosomal phosphatidylinositol 3,4-bisphosphate. Science 2017;356:968–972. 28572395 10.1126/science.aaf8310

[14] Rodriguez-Escudero I, Oliver MD, Andres-Pons A, Molina M, Cid VJ, Pulido R. A comprehensive functional analysis of PTEN mutations: implications in tumor- and autism-related syndromes. Hum Mol Genet 2011;20:4132–4142. 21828076 10.1093/hmg/ddr337

[15] Malek M, Kielkowska A, Chessa T, et al. PTEN regulates PI(3,4)P2 signaling downstream of class I PI3K. Mol Cel 2017;68:566–580. 10.1016/j.molcel.2017.09.024 PMC5678281 29056325

[16] Olney RK, Lewis RA, Putnam TD, Campellone JV. Consensus criteria for the diagnosis of multifocal motor neuropathy. Muscle Nerve 2003;27:117–121. 12508306 10.1002/mus.10317

[17] Cottenie E, Menezes MP, Rossor AM, et al. Rapidly progressive asymmetrical weakness in Charcot-Marie-Tooth disease type 4J resembles chronic inflammatory demyelinating polyneuropathy. Neuromuscul Disord 2013;23:399–403. 23489662 10.1016/j.nmd.2013.01.010

[18] Mensah LB, Davison C, Fan SJ, Morris JF, Goberdhan DC, Wilson C. Fine-tuning of PI3K/AKT signalling by the tumour suppressor PTEN is required for maintenance of flight muscle function and mitochondrial integrity in ageing adult Drosophila melanogaster. PLoS One 2015;10:e0143818. 26599788 10.1371/journal.pone.0143818 PMC4658134

[19] Sarker D, Ang JE, Baird R, et al. First-in-human phase I study of pictilisib (GDC-0941), a potent pan-class I phosphatidylinositol-3-kinase (PI3K) inhibitor, in patients with advanced solid tumors. Clin Cancer Res 2015;21:77–86. 25370471 10.1158/1078-0432.CCR-14-0947 PMC4287394

[20] Ang JE, Pandher R, Ang JC, et al. Plasma metabolomic changes following PI3K inhibition as pharmacodynamic biomarkers: preclinical discovery to phase I trial evaluation. Mol Cancer Ther 2016;15:1412–1424. 27048952 10.1158/1535-7163.MCT-15-0815 PMC5321508

[21] Ren Y, Hao P, Law SK, Sze SK. Hypoxia-induced changes to integrin alpha 3 glycosylation facilitate invasion in epidermoid carcinoma cell line A431. Mol Cell Proteomics 2014;13:3126–3137. 25078904 10.1074/mcp.M114.038505 PMC4223496

[22] Dey N, Crosswell HE, De P, et al. The protein phosphatase activity of PTEN regulates SRC family kinases and controls glioma migration. Cancer Res 2008;68:1862–1871. 18339867 10.1158/0008-5472.CAN-07-1182

